# Micro, meso, macro factors that exacerbate or alleviate the social isolation of racially and ethnically diverse older adults living alone with cognitive impairment in the United States

**DOI:** 10.1002/alz.095586

**Published:** 2025-01-09

**Authors:** Elena Portacolone, Thi Tran, Daisy Feddoes, Andrew Cohen

**Affiliations:** ^1^ University of California San Francisco, San Francisco, CA USA; ^2^ Philip Lee Institute on Health Policy Studies, San Francisco, CA USA; ^3^ Yale School of Medicine, New Haven, CT USA

## Abstract

**Background:**

One out of four older adults with cognitive impairment in the United States are estimated to live alone, about 4.3 million racially and ethnically diverse individuals. Because living alone often signals limited social supports and a desire for privacy and independence, older adults living alone with cognitive impairment are at elevated risk for social isolation, which is a risk factor for adverse events. A key gap in knowledge is that we do not know what specific factors either exacerbate or alleviate the social isolation of this population. To address this gap, we used the micro, meso, and macro framework to identify these factors in a diverse sample of older adults living alone with cognitive impairment.

**Method:**

This qualitative study of older adults living alone with cognitive impairment used semi‐structured interviews conducted between June 2022, and February 2024, with purposively sampled study participants in Michigan, California, and Louisiana. An inductive and deductive content analysis was used to analyze the interview transcripts.

**Result:**

We conducted 39 interviews with 10 older adults living alone with cognitive impairment (mean [SD] age, 74.1 [11.0] years); all were female, 4 self‐identified as Chinese American, 3 as Black/African American, 2 as Hispanic/Latino and 1 as white. All Chinese American participants were monolingual Mandarin; one Hispanic/Latino participants was monolingual Spanish. Three participants were rural residents. A key factor that exacerbated isolation was a strong preference for remaining inside the home because of its familiarity. Other exacerbating factors included deaths/losses of family members and significant others, hiding feelings, and having members of the social circle disconnecting from older adults living alone with cognitive impairment. Conversely, the most prominent factors that prevented isolation was having strong relationships with proactive family members and belonging to religious groups. Other factors included enduring difficult relationships and attending structured activities.

**Conclusion:**

Most factors that either alleviated or exacerbated the social isolation of older adults living alone with cognitive impairment pertained to their personal sphere. As a result, effective policies and programs to address isolation in this population should focus on reinforcing family connections, strengthening structured activities, and supporting attendance of religious and other meaningful groups.

